# High-Level Expression and Biochemical Properties of A Thermo-Alkaline Pectate Lyase From *Bacillus* sp. RN1 in *Pichia pastoris* With Potential in Ramie Degumming

**DOI:** 10.3389/fbioe.2020.00850

**Published:** 2020-07-24

**Authors:** Xueyun Zheng, Yimin Zhang, Xiaoxiao Liu, Cheng Li, Ying Lin, Shuli Liang

**Affiliations:** ^1^Guangdong Provincial Key Laboratory of Fermentation and Enzyme Engineering, South China University of Technology, Guangzhou, China; ^2^Guangdong Research Center of Industrial Enzyme and Green Manufacturing Technology, School of Biology and Biological Engineering, South China University of Technology, Guangzhou, China; ^3^Department of Biology, Massachusetts Institute of Technology, Cambridge, MA, United States

**Keywords:** pectate lyase, high-level expression, biochemical properties, ramie degumming, *Pichia pastoris*

## Abstract

Pectate lyases play an essential role in textiles, animal feed, and oil extraction industries. *Pichia pastor*is can be an ideal platform for pectate lyases production, and BspPel (a thermo-alkaline pectate lyase from *Bacillus* sp. RN1) was overexpressed by combined strategies, reaching 1859 U/mL in a 50 L fermentator. It displayed the highest activity at 80°C, and maintained more than 60% of the activity between 30 and 70°C for 1 h. It showed an optimal pH of 10.0, and exhibited remarkable stability over a wider pH range (3.0-11.0), retaining more than 80.0% of enzyme activity for 4 h. The *K*_*m*_ and *k*_*cat*_ of BspPel on PGA (polygalacturonic acid) was 2.19 g L^–1^ and 116.1 s^–1^, respectively. The activity was significantly enhanced by Ca^2+^, Mn^2+^, and Cu^2+^, and a slight increase was observed with the addition of Ba^2+^ and Mg^2+^. Scanning electron microscope was used to show the degumming efficiency of BspPel on ramie fibers. The loss weight was 9.2% when treated with crude enzyme supernatant and 20.8% when treated with the enzyme-chemical method, which was higher than the 14.2% weight loss in the positive control treated with 0.5% (w/v) NaOH alone. In conclusion, BspPel could be a good candidate for the ramie degumming industry.

## Introduction

Pectin, an ubiquitous structure constituent of the middle lamella and the primary cell wall in the plant (Soriano et al., 2006), is a heteropolysaccharide composed essentially of long chains of α-1,4-D-polygalacturonate, which are partially methyl esterified (Van Truong et al., 2001). Ramie (*Boehmeria nivea*) produces one of the strongest and longest plant fibers, which are used for industrial packaging, clothing fabrics, twines, cordages, car outfits, etc. (Hester and Yuen, 1989). However, ramie fibers have gum material (20–35%), which contains pectin and hemicellulose, hindering its application. To achieve widespread application, it is important to remove the pectin when processing the fiber.

Pel (Pectate lyase; pectate transeliminase, EC 4.2.2.2) is the pectin depolymerizing enzyme that cleaves α-1,4- galacturonosidic linkages of PGA (polygalacturonic acid), a key component in pectin, and generates an unsaturated bond between C4 and C5 of the newly formed oligogalacturonide at the non-reducing end via a trans-elimination mechanism (Klug-Santner et al., 2006). The enzymatic degradation of pectin requires the combined action of several pectinolytic enzymes with different methods of action (Jayani et al., 2005), and this enzymatic methods can efficiently remove ramie gum under mild and flexible conditions and serve as a good alternative to conventional degumming methods, with decreased environmental pollution, less damage to the fibers and improved yield and quality of degummed ramie (Zhang et al., 2013; Zhou et al., 2017a). In addition to various environmentally friendly and economic applications in industrial sectors (Kohli and Gupta, 2015), Pels have also been used in tea and coffee fermentation (Godfrey, 1998), animal feed (Combo et al., 2012), and oil extraction (Kashayp et al., 2001), indicating their potential use in the food and feed industries. Among pectate lyases, alkaline Pels are desired for removing the pectin from ramie, since pectin is more soluble in alkaline solution.

Comprehensive studies on alkaline Pels have been carried out for many years. The chief source of acidic Pels is fungi, but alkaline Pels are produced from alkalophilic bacteria, primarily *Bacillus* spp. (Kavuthodi and Sebastian, 2018). These enzymes showed good performance in bioscouring and degumming applications. However, due to low activity and stability under processing conditions, few Pels were found to be economically efficient for industrial applications (Sukhumsiirchart et al., 2009; Liang et al., 2015). Therefore, it is important to find new enzymes that can effectively function at moderate temperature [40–70°C] and alkaline pH (Kashayp et al., 2001; Kohli and Gupta, 2015). Pels with higher activity and stability under the industrial processing conditions are still in great demand.

Many pectate lyases are cloned and expressed in *Escherichia coli* (*E. coli*). However, this is unfavorable for large-scale production due to the requirement of expensive IPTG (isopropyl thiogalactoside) as an inducer. Meanwhile, *Bacillus* is not a suitable host for pectate lyases during the degumming process since most *Bacillus* produce cellulases endogenously with a detrimental effect on the fiber (Zhang et al., 2013). Research to improve enzyme productivity using a high-level expression platform is required to meet industrial applications. The methylotrophic yeast *Pichia pastoris (P. pastoris)* is considered an excellent expression system for the production of recombinant heterologous proteins obtained from different sources (Čiplys et al., 2015). The popularity of this expression system is due to its many advantages, such as the simplicity of the techniques needed for genetic manipulation, the efficient expression and secretion capacity, a high growth rate, moderate post-translational modifications, simple nutrient utilization and suitability for high-density fermentation. *P. pastoris*, which does not express endogenous cellulases and has high secretion capability, will be an ideal host for pectate lyases expression (Zhang et al., 2013).

In the present study, a thermo-alkaline pectate lyase (BspPel) from *Bacillus* sp. RN1 was overexpressed in *P. pastoris* with combined strategies, and the biochemical characteristics of recombinant BspPel were studied. Additionally, the potential application of BspPel in the ramie fiber degumming industry was further evaluated.

## Results and Discussion

### Functional Expression of BspPel in *P. pastoris*

**FIGURE 1:**
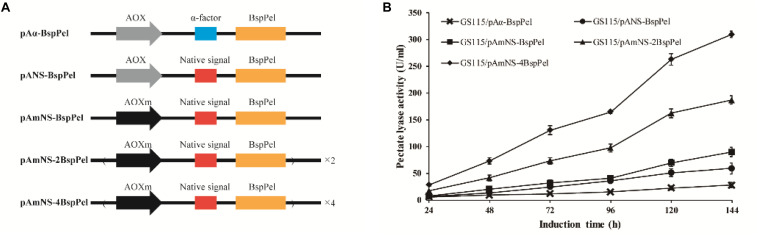
Functional expression of BspPel in *P. pastoris*. **(A)** Schematic diagrams of recombinant vectors. AOX: AOX1 promoter, AOXm: modified AOX1 promoter, α-factor: α-factor signal peptide, Native signal: native signal peptide; **(B)** Time course of BspPel expression in recombinant strains.

The thermo-alkaline Pel from *Bacillus* sp. RN1 (GenBank Accession No. AB428424) showed high homology to the characterized Pel from *Bacillus licheniformis* S91 (GenBank no. ANS59493.1) (Zhou et al., 2017a) with 98% identity (see Supplementary Figure 1), indicating that BspPel also belongs to the PL1 family. The native signal of the *BspPel* gene was predicted by SignalP 4.1 Server^1^, and the corresponding sequences was: ATGAAGAAGTTGATTAGTATTATTTTTATTTTTGTTTTGG GAGTTGTTGGTTCCTTGACCGCAGCAGTTAGTGCC. The *S. cerevisiae (Saccharomyces cerevisiae)* α-factor prepro-peptide is the most commonly used signal sequence for mediating effective protein secretion. Therefore, BspPel without native signal peptide sequence was cloned into the *P. pastoris* expression vector pPICHKA, which has a α-factor prepro-peptide, generating the plasmid pPICHKA-BspPel (pAα-BspPel), which harbored the commonly used AOX1 promoter and α-factor signal peptide (Figure 1A). The enzyme activity of BspPel expressed in GS115/pAα-BspPel using shake-flask cultivation reached approximately 28.0 U/mL under standard assay conditions with PGA as a substrate after 144 h of induction (Figure1B).

**FIGURE 2:**
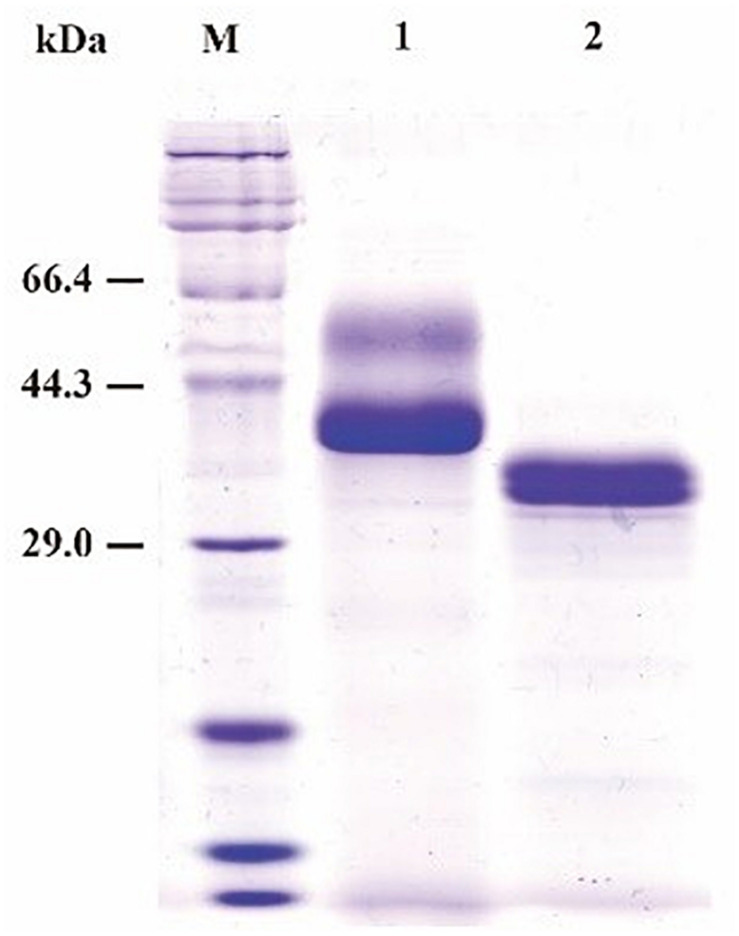
SDS-PAGE assay of the recombinant BspPel. Lane M: protein marker; lane 1: purified recombinant BspPel; lane 2: purified recombinant BspPel treated by PNGase F.

SDS-PAGE (sodium dodecyl sulfate polyacrylamide gel electrophoresis) analysis showed that the purified recombinant BspPel had two bands with molecular masses of 56 and 37 kDa as shown in lane 1 (Figure 2), both larger than predicted. After a deglycosylation step by PNGase F, only one 33 kDa band with the same size as the one expressed in the natural host *Bacillus* sp. RN1 (Sukhumsiirchart et al., 2009) was observed in lane 2 (Figure 2), suggesting that the recombinant BspPel had different degrees of glycosylation. Three potential N-glycosylation consensus sites (Asn89, Asn109, and Asn217) (see Supplementary Figure 1) were found using the NetNGlyc analysis tool, which may indicate different extents of glycosylation and larger molecular weights.

### Enhancement of BspPel Expression Through Combined Strategies

Although the *S. cerevisiae* α-factor prepro-peptide can be used to express BspPel, the potential of native signal peptides of BspPel in this production was also investigated, since native signal peptides from several proteins, such as human calreticulin (Čiplys et al., 2015), *Candida antartica* lipase B (Vadhana et al., 2013), *Trichoderma reesei* Cel61A (Tanghe et al., 2015) and so on, were found to outperform the α-factor. In this study, the whole gene containing the potential native signal peptide with 25 amino acids was used for direct expression, generating the pANS-BspPel vector to compare the effects of α-factor prepro-peptide and native signal peptide on BspPel production. As shown in Figure 1B, the strain GS115/pANS-BspPel expressing the enzyme with native prepro-peptide achieved 59.2 U/mL, which markedly increased by 1.14-fold compared to the strain called GS115/pAα-BspPel with α-factor, suggesting that the secretion efficiency of native signal peptide was significantly higher than that of α-factor.

The expression of a recombinant protein always depends on its transcription level, and therefore, a strong promoter is necessary for high-level expression. The AOX1 promoter is the most commonly used promoter in the *P. pastoris* expression platform, and the use of modified AOX1 promoters (AOX_m_) resulted in an increase of promoter activity by approximately 30% (Hartner et al., 2008). The expression of BspPel in the recombinant strain GS115/pAmNS-BspPel increased by 51.5% compared to that in the GS115/pANS-BspPel strain and exhibited approximately 89.7 U/mL activity after methanol induction for 144 h (Figure 1B).

Increasing gene copy number is another effective strategy for improving protein expression. Repeat insertion of the expression cassette (AOXm-NS-BspPel) was performed, and plasmids pAmNS-2BspPel and pAmNS-4BspPel were obtained, which contained two and four copies of BspPel, respectively. qPCR assays were performed to precisely determine the *BspPel* gene copy number in all recombinant strains. Prtp1/Prtp2, and Prtg1/Prtg2 were used as primers to conduct qPCR for gene copy number of *BspPel* in genomic DNA. The sequences of primers are shown in Table 1. The *BspPel* gene copy numbers of strains GS115/pANS-BspPel, GS115/pAmNS-BspPel, GS115/pAmNS-2BspPel, and GS115/pAmNS-4BspPel, normalized to the reference *GAPDH* (glyceraldehyde-3-phosphate dehydrogenase) gene fragment, were 1.01, 1.06, 2.03, and 4.00, respectively. For the strains with multiple copies of the *BspPel* gene, the BspPel activity of GS115/pAmNS-2BspPel was 187 U/mL, and the activity was further improved to 310 U/mL in GS115/pAmNS-4BspPel, representing an approximately 11.1-folds increase compared to the activity of the original strain GS115/pAα-BspPel. The highest BspPel activity was obtained when the copy number of the *BspPel* gene reached 4, indicating that increasing gene dosage enhanced protein yield. As far as we know, the enzyme activity was just lower than that of BacPelA, which reached 490.2 U/mL in *E. coli* (Zhou et al., 2017b). There are also some restrictions that hinder further production. For example, a plateau effect occurred when the gene dosage exceeded a certain range due to metabolic burden (Gasser et al., 2008; Li et al., 2015). Consequently, engineering of protein folding, ERAD (endoplasmic reticulum-associated protein degradation) systems and transport systems should be performed to further improve protein production (Zahrl et al., 2017). There is still huge potential to achieve higher production with *P. pastoris.*

**TABLE 1:** Strains, plasmids and oligonucleotides used in this study.

Strains, plasmids and oligonucleotides	Genotype and characteristics	References
**Strains**		
*E. coli* TOP10		Invitrogen
*P. pastoris* GS115	*HIS4 Mut^+^His^–^(AOX1^+^, AOX2^+^)*	Invitrogen
GS115/HKA	*Mut^+^His^+^ (AOX1^+^, AOX2^+^)*, pPICHKA transformants	Lab stock
GS115/pAα-BspPel	Mut^+^ His^+^ *(AOX1^+^, AOX2^+^)*, pAα-BspPel transformants	This study
GS115/pANS-BspPel	Mut^+^ His^+^ *(AOX1^+^, AOX2^+^)*, pANS-BspPel transformants	This study
GS115/pAmNS-BspPel	Mut^+^ His^+^ *(AOX1^+^, AOX2^+^)*, pAmNS-BspPel transformants	This study
GS115/pAmNS-2BspPel	Mut^+^ His^+^ *(AOX1^+^, AOX2^+^)*, pAmNS-2BspPel transformants	This study
GS115/pAmNS-4BspPel	Mut^+^ His^+^ *(AOX1^+^, AOX2^+^)*, pAmNS-4BspPel transformants	This study
**Plasmids**		
pPICHKA	*HIS4 Kan^*r*^* AOX1 promoter	Lab stock
pAα-BspPel	*HIS4 Kan^*r*^* AOX1 promoter	This study
pANS-BspPel	*HIS4 Kan^*r*^* AOX1 promoter	This study
pAmNS-BspPel	*HIS4 Kan^*r*^* AOXm promoter	This study
pAmNS-2BspPel	*HIS4 Kan^*r*^* AOXm promoter	This study
pAmNS-4BspPel	*HIS4 Kan^*r*^* AOXm promoter	This study
**Oligonucleotides(5′ → 3′)**		
BspPel-s1	GGCGCGGCGAATTCATGAAGAAGTTGATTAGT	This study
BspPel-s2	ATTTGCGGCCGCTTTAAGGGTTGATTTTTCC	This study
BspPel-s3	TCAGAATTCGAAGCCGCCTCTGCTTTGAAC	This study
Prtp1	ACTACAGGTGGAGAGGGTGGAC	This study
Prtp2	GACGATTGAAACATTGGAGACG	This study
Prtg1	GTCGGGACACGCCTGAAACT	This study
Prtg2	CCACCTTTTGGACCCTATTGAC	This study

*The restriction enzyme sites are underlined.*

### Scale-Up BspPel Production in a 50 L Fermenter

**FIGURE 3:**
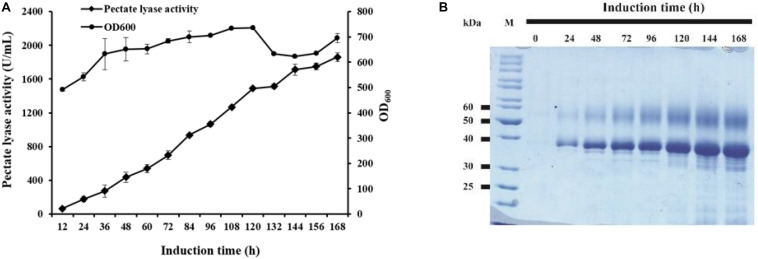
The time course of recombinant enzyme production in a 50 L fermentator and SDS-PAGE analysis. **(A)** Time dependence of the activity of pectate lyase and cell growth of GS115/pAmNS-4BspPel after induction with methanol, using GS115/HKA as the background (control) sample; **(B)** SDS-PAGE assay of the collected culture supernatant from the most effective strains in the fermentator after 168 h of induction with methanol. Lane M: protein marker; lane 1–lane 8: recombinant protein expression from 0 to 168 h.

The strain GS115/pAmNS-4BspPel with the highest secretion level was further evaluated by high-density fermentation in a 50 L fermenter. At the end of the glycerol fed-batch phase, the OD_600_ reached 476, and methanol feeding started to induce BspPel expression. After 168 h of induction, the enzyme activity in the culture medium reached approximately 1859 U/mL, which was six-fold of that in shake-flask cultivation (Figure 3A).

The expressed proteins in the supernatant were visualized by Coomassie brilliant blue staining after SDS-PAGE of the culture supernatants (Figure 3B). The molecular weight and glycosylation properties of BspPel expressed in the 50 L bioreactor were identical to those in the shake flask. The protein concentration increased with increasing induction time and reached 9.5 g/L after 168 h of induction.

### Biochemical Properties of BspPel

**FIGURE 4:**
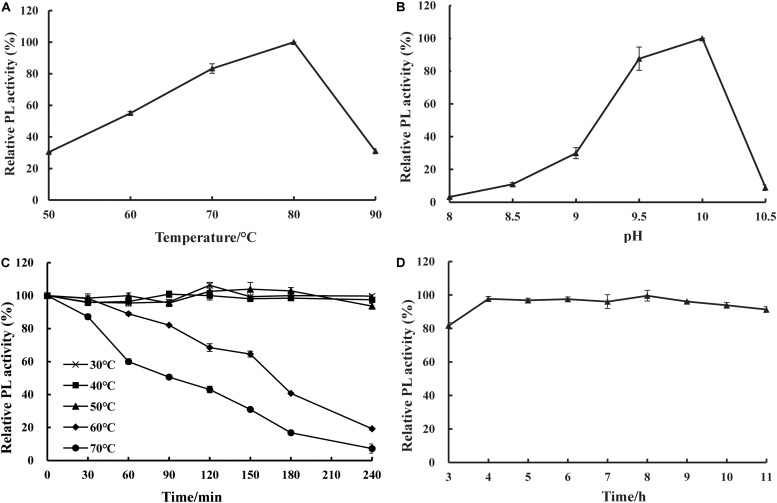
Effect of **(A)** temperature, **(B)** pH on purified pectate lyase (PL) activity, **(C)** temperature stability and **(D)** pH stability. Values are averages of results from triplicate trials; error bars indicate the SD values.

The effects of temperature and pH on the activity and stability of purified recombinant BspPel were determined using PGA as a substrate. As shown in Figures 4A,B, the optimal temperature was 80°C, and the optimum pH was 10.0. The thermal stability assay showed that BspPel was remarkably stable at 30–50°C, and no loss in activity was detected after 4 h of incubation (Figure 4C). Since a moderate temperature (37–55°C) is used in bioresource processing, and enzymatic processes are often carried out at 45 or 50°C (Li et al., 2014), BspPel was tested in such a temperature range for 4 h and showed more than 93% activity, indicating great stability. When the enzyme was cultivated at 60 and 70°C for 30 min, there was still >98 and 87% activity, respectively. More than 60% of the activity was maintained in the range of 30–70°C for 1 h (more than 80% of the activity was maintained in the range of 30–60°C for 1 h). BspPel showed higher activity than many enzymes such as PEL68P (Zhang et al., 2013), PL (Zhuge et al., 2008), Pectate lyase (Chiliveri and Linga, 2014), BsPel-PB1 (Zhou et al., 2017c), and PpPel10a (Zhao et al., 2018) when treated at 70°C. To our best knowledge, only Pel-20 (Saoudi et al., 2015) and Pel SWU (Sukhumsiirchart et al., 2009) retained more activity than BspPel at 70°C, but BspPel was treated with a higher pH, which had important effects on activity. The residual activity of BspPel was reduced to approximately 19% and 7% after incubation at 60°C and 70°C for 4 h, respectively. Even though BliPelA (Zhou et al., 2017a) had 98% identity with BspPel in protein sequence, they had different thermostability. While BliPelA obtained 60% of activity under incubation at 65°C for 30 min at pH 9, BspPel treated in 70°C showed higher activity at higher pH. Glycosylation confers BspPel stability against themal inactivation and the comformational and/or dynamic properties of a glycosylated proteins are likely to differ from its unglycosylatd counterpart (Guo et al., 2008; Zhou et al., 2017b). BspPel had an optimum pH under alkaline conditions and was stable over a wider acid-to-alkaline pH range (3.0–11.0). In this pH range, more than 80.0% of the original enzyme activity was retained after 16 h at 25°C (Figure 4D). Due to its alkaline pH tolerance, BspPel should provide a potential advantage in the kraft-pulp and detergent manufacturing industries, which require the use of high pH.

The effect of several metal ions and chemicals on the activity of BspPel were evaluated. The activity of BspPel was obviously increased by Ca^2+^ as shown in Table 2, and the highest activity was observed with the addition of 0.5 mM CaCl_2_ (see Supplementary Figure 2). Compared to control without metal ions, BspPel activity was almost five-fold higher when Ca^2+^ was added. BspPel activity was notably activated by Mn^2+^ and Cu^2+^, and a slight increase was observed with the addition of Ba^2+^ at concentrations of 0.5 or 1 mM. In particular, a relative activity of 194% was detected with Mn^2+^ at a concentration of 1 mM. Most Pels were susceptible to divalent cations, and the activity of BspPel was significantly activated by Cu^2+^ and Mn^2+^, similar to the BliPelA from *B. licheniformis* (Zhou et al., 2017a). In contrast, Pel from *B. subtilis* was inhibited strongly by Cu^2+^, Mn^2+^ and Hg^2+^ (Zhang et al., 2013). BspPel might substitute Cu^2+^ and Mn^2+^ for Ca^2+^ at the catalytic site, and further mutagenesis analyses at the Ca^2+^-binding site should be performed to clarify the catalytic role of these ions. More importantly, the addition of these two ions may have positive effects on the degumming process in the textile industry. Besides, BspPel activity was completely inhibited by EDTA (1 mM) and partly inhibited by Zn^2+^, Ni^2+^, K^+^, and Li^+^. SDS at a concentration of 0.5 mM had a mild negative influence on BspPel activity, while severe inhibition was detected when the concentration was increased to 1 mM. However, BspPel still showed higher tolerance to SDS, with more than 72 and 24% activity retained following incubation at the concentrations of 0.1 and 0.5%, respectively, than most previously reported Pels, which lost nearly all activity (Zhang et al., 2013; Zhou et al., 2017a, b). Similar to the residues of BliPelA, the residues R233 and R238/K204 of BspPel are conserved and play a comparable role as the essential catalytic bases, and the three aspartate residues D150, D180, and D184 are completely conserved in BspPel for Ca^2+^ interaction (Zhou et al., 2017a). In general, Ca^2+^ is required for the pectolytic activity of most Pels.

**TABLE 2:** Effects of different chemicals on the activity of BspPel.

Chemicals	Concentration	Relative activity (%)*	Concentration	Relative activity (%)*
Control	0	100.00	1 mM	100.00 ± 0
CaCl_2_	0.50 mM	496.06 ± 0.01	1 mM	448.12 ± 0.03
MgCl_2_	0.50 mM	99.77 ± 4.51	1 mM	109.72 ± 3.19
BaCl_2_	0.50 mM	115.58 ± 2.74	1 mM	119.60 ± 7.69
NiCl_2_	0.50 mM	98.78 ± 3.07	1 mM	86.30 ± 2.75
MnSO_4_	0.50 mM	136.23 ± 5.80	1 mM	154.15 ± 6.34
CuSO_4_	0.50 mM	170.18 ± 5.21	1 mM	194.68 ± 5.13
KCl	0.50 mM	88.98 ± 4.94	1 mM	83.02 ± 2.41
LiCl	0.50 mM	94.44 ± 4.62	1 mM	91.27 ± 1.48
ZnCl_2_	0.50 mM	88.55 ± 1.45	1 mM	85.29 ± 5.62
EDTA	0.50 mM	22.09 ± 2.79	1 mM	3.82 ± 0.12
SDS	0.10%	72.89 ± 2.11	0.50%	24.27 ± 1.79

* The activity under standard conditions was used as the reference (100%), and data represent means ± SD (n = 3) relative to the untreated reference samples.

**FIGURE 5:**
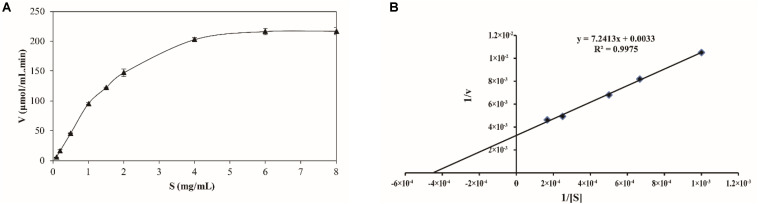
**(A)** Substrate-velocity curve and **(B)** Lineweaver–Burk plot of purified BspPel. Values are averages of results from triplicate trials; error bars indicate the SD values.

The kinetics of recombinant BspPel were analyzed using PGA as a substrate. The reaction was performed in a 50 mM Gly-NaOH buffer (pH 10.0) at 80°C with PGA concentrations from 0.1 to 8 mg mL^–1^ (Figure 5A). Lineweaver–Burk plots (Figure 5B) were used to calculate the kinetic parameters. The corresponding *V*_*max*_ and *k*_*cat*_ of BspPel on PGA were 0.30 mol L^–1^ min^–1^ and 116.1 s^–1^, respectively. The affinity of BspPel, indicated by *K*_*m*_, was 2.19 g L^–1^, and the catalytic efficiency *K*_*cat*_/*K*_*m*_ was 53 L s^–1^ g^–1^.

The substrate specificity of BspPel was determined using PGA and various pectins (Sigma-Aldrich). Specific activities were determined as 106.01 ± 0.72 and 127.88 ± 0.87 U/mg for PGA and Pectin (55–70% methylated). BspPel showed 120.63% activity on pectin (55–70% methylated) relative to PGA (taken as 100.0%), and thus, pectin with the methylation degree of 55–70% appears to be a preferred substrate over PGA, whereas no activity was detected on pectin from apples or citrus peels (Table 3). The substrate specificity of BspPel contrasted that of BacPelA (Zhou et al., 2017b), BliPelA (Zhou et al., 2017a) and Pel-7 (Kobayashi et al., 1999) which exhibited lower activity toward medium-methylated pectins. For example, specific activities of BliPelA were 320 and 153 U/mg for PGA and pectin (55–70% methylated). BliPelA only showed 47.8% activity on pectin (55–70% methylated) relative to PGA (taken as 100.0%), indicating PGA would be a desirable substrate for it. A protein secondary structure prediction server-Jpred4 (Drozdetskiy et al., 2015) was used to predict the BspPel protein, finding that the one (Asn89) of three glycosylation sites was located in the β-sheet where there may be a change in secondary structure. Meanwhile, there may be some specific or different features on the 3D structure, especially at the substrate-binding and catalytic domains, generating the unique properties of Pels on different substrates (Zhou et al., 2017b). For BspPel, the higher catalytic activity for pectins with medium degrees of methylation suggests it may be a potential candidate for industrial application in ramie degumming because the methylated degree of PGA chains in ramie fibers is often as high as 60–75% (Calafell et al., 2005).

**TABLE 3:** Substrate specificity of BspPel.

Substrate	Specific activity (U/mg)^a^	Relative activity (%)
PGA	106.01 ± 0.72	100.00 ± 0
Pectin, 55–70% methylated	127.88 ± 0.87	120.63 ± 0.95
Pectin (from apples)	nd	nd
Pectin (from citrus peels)	nd	nd

*nd: no activity was detected. ^a^The activity was determined by the A235 method, and the values are expressed as the averages of three experiments.*

### Degumming of Ramie Fiber by BspPel

**FIGURE 6:**
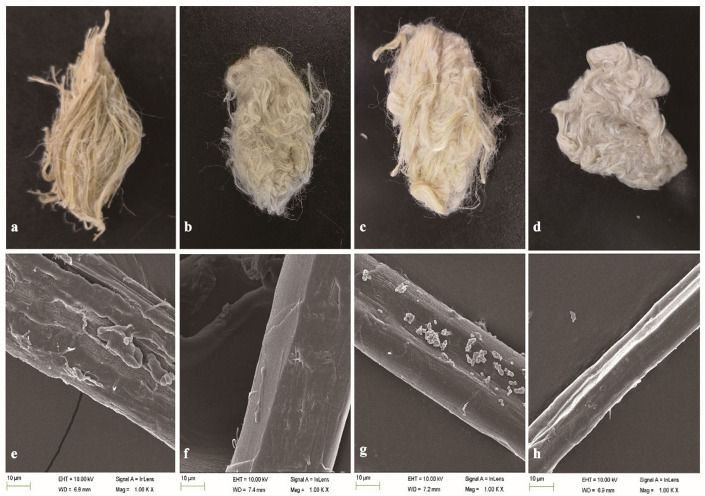
SEM images of ramie fibers treated **(a,e)**: with buffer only (negative control); **(b,f)**: with BspPel; **(c,g)**: with chemicals only (positive control); and **(d,h)**: with the enzyme-chemical method. **(a–d)**: External images of ramie fibers; **(e–h)**: surface images of single fibers (1000 × magnification).

The weight loss of ramie fibers without boiling treated with BspPel was 9.2%, while 14.2% weight loss was obtained for the positive control (chemicals). For the enzyme-chemical treatment, the weight loss was 20.8%, similar to BspPelA (Zhou et al., 2015), which was higher than that of the positive control treated with 0.5% (w/v) NaOH alone, indicating that even if the fibers were not processed by boliling, pectin was still detectably removed. Morphologically, the enzyme-chemical treated ramie fibers were softer, whiter and more dispersed than fibers treated with alkaline buffer or chemicals alone (Figures 6a–d). Moreover, electron microscopic images of single fibers showed that the surface of the enzyme-chemical treated ramie fibers was smoother than those treated with alkaline buffer or chemicals (Figures 6e–h).

The use of several alkaline and thermostable Pels has been reported in the ramie degumming process. For instance, weight loss of 13.5, 21.5, 23.1, and 24.8% was observed after 4 h of degumming by Pels from *B. subtilis* 7-3-3 (Zou et al., 2013), *B. licheniformis* 91 (Zhou et al., 2017a), *B. pumilus* ATCC7061 (Liang et al., 2015) and *B. clausii* S10 (Zhou et al., 2017b), respectively. In addition, treatment with Pels from *Amycolata sp.* (Brühlmann et al., 2000) and *B. pumilus* DKS1 (Basu et al., 2011) resulted in approximately 16.7 and 17% weight loss after 15 h and 24 h of degumming. These dugumming processes were conducted in slightly different conditions, like higher enzyme activity, temperature or longer time, compared to the present study. In this study, the degumming efficiency via the combined enzyme-chemical process achieved weight loss of 20.8% under a moderate condition after 4 h, which is comparable with the reported results, indicating BspPel was an efficient candidate for bio-degumming. Moreover, optimizing the operating conditions and combining the utilization with chemicals or other pectin-degrading enzymes could be possible to further improve the degumming efficiency.

## Materials and Methods

### Strains, Plasmids, and Media

*Escherichia coli* TOP10 (Invitrogen, Carlsbad, CA, United States) was used for DNA manipulation, gene cloning and sequencing. *E. coli* transformants were selected on LB medium plates (Swords, 2003) containing 50 μg/mL kanamycin or low-salt LB medium plates with decreased salt concentration of 0.5% containing 25 μg/mL Zeocin. *P. pastoris* strain GS115 (ATCC 20864) and vectors pPIC9K and pPICZαA were purchased from Invitrogen Co. (Invitrogen, United States). GS115 and recombinant *P. pastoris* strains were cultivated in either YPD (yeast extract peptone dextrose) (Weidner et al., 2010), BMGY (buffered complex glycerol) (Weidner et al., 2010) or BMMY (buffered complex methanol medium). BMMY was the same as BMGY, except 1% methanol replaced glycerol. The transformed *P. pastoris* were selected on MD plates (Higgins, 1995). Strains, vectors and primers used in this study AOX1 summarized in Table 1.

### Construction of Expression Plasmids

The *BspPel* gene (GenBank Accession No. AB428424) wascodon-optimized and amplified using the primer pairs BspPel-s1, BspPel-s2 and BspPel-s3, BspPel-s2. The *Bacillus* sp. RN1 pectate lyase (BspPel) was expressed with a native leader sequence/α-factor (originated from *S. cerevisiae*) signal sequence and tagged with 6× His on the C-terminus. The PCR product was separated by electrophoresis and the fragment was then purified with a QIAquick Gel Purification Kit. Both of the fragments and vector pPICHKA (a plasmid which is generated by pPICZαA combined *HIS4* gene from pPIC9k) were digested with *Eco*RI and *Not*I, and they were then ligated by a DNA Ligation Kit. The resulting products pPICHKA-AOX1-S1-BspPel (pANS-BspPel) with the BspPel native signal peptide (S1), which was predicted by software SignalP 4.1 Server^1^, and pPICHKA-AOX1-α-BspPel (pAα-BspPel) with α-factor secretion signal peptide of *S. cerevisiae* were transformed into *E. coli* TOP10. Positive recombinant strains were screened via PCR. We also reconstructed the AOX_m_ promoter according to the promoter library (Vadhana et al., 2013) to produce the vector pPICHKA-AOX_m_-S1-BspPel (pA_m_NS-BspPel). The restriction enzymes *Bgl*II and *Bam*HI were used to digest pA_m_NS-BspPel to obtain the expression cassette (containing the promoter, BspPel and terminator), as the two restriction enzymes have the same cohesive ends. After the linearization of pA_m_NS-BspPel with *BamH*I, the expression cassette and the plasmid were ligated using a DNA Ligation Kit to obtain the two-copy plasmid pPICHKA-AOX_m_-S1-BspPel-2copy (pA_m_NS-2BspPel). The same method was used to obtain four-copy plasmid pPICHKA-AOX_m_-S1-BspPel-4copy (pA_m_NS-4BspPel) (Figure 1B). *Bsp*EI was used to linearize pA_m_NS-BspPel, pA_m_NS-2BspPel, pA_m_NS-4BspPel for transformation into *P. pastoris* GS115. The transformants were screened on MD plates, and the recombinant strains were verified by colony PCR. PrimerSTAR^TM^ HS DNA Polymerase, DNA ligation kit ver. 2.0 and restriction endonucleases were purchased from TaKaRa Biotechnology Co. (Dalian, China). All recombinant plasmids were confirmed by DNA sequencing by Sangon Biotechnology Co. (Shanghai, China), and all primers used in this study are listed in Table 1.

### *P. pastoris* Transformation

The recombinant plasmids pAα-BspPel, pANS-BspPel, pA_m_NS-BspPel, pA_m_NS-2BspPel, and pA_m_NS-4BspPel were linearized with *Bsp*EI, for which the restriction site was located in the *HIS4* sequence, and transformed into *P. pastoris* GS115 competent cells by electroporation with a Gene Pulser apparatus (Bio-Rad, Hercules, CA, United States) according to the manufacturer’s protocol. Transformants were selected on MD plates after incubation at 30°C for 2–3 days.

### Determination of Gene Copy Number by Quantitative PCR

Absolute quantification was used to determine the copy numbers of the target genes using SYBR green. The plasmid pPICHKA-BspPel-G, consisting of a portion of the *GAPDH* gene and a following genomic sequence, was used as the reference, since there is only a single copy in the *P. pastoris* genome. Genomic DNA was extracted from *P. pastoris* using the Yeast DNAiso-Kit (Takara, Shiga, Japan) according to the manufacturer’s manual. Prtp1/Prtp2 and Prtg1/Prtg2 were used as primers at a concentrations of 400 nM with genomic DNA as the template to conduct qPCR to detect the gene copy number of *BspPel* in genomic DNA. Primer design was performed using Primer Express v3.0 software (Applied Biosystems, CA, United States), and all primers used for qPCR are listed in Table 1. According to the product manual, PCR was performed in a 10 μL reaction mixture containing 5 μL of 2× SuperReal PreMix Plus (TIANGEN BIOTECH Co., Beijing, China), 0.3 μL of each 10 μM primer, 1 μL of 50× ROX Reference Dye, and 1 μL of template. Real-time PCR was performed using an ABI 7900HT system with SDS v2.4 Software (Applied Biosystems). A tenfold dilution series (10^–2^–10^–8^) of linearized plasmid, including cloned target and reference genes, was utilized as templates to establish the standard curves. Data were collected, and the copy number of the target gene was analyzed in different recombinant strains. The copy number *BspPel* in each transformant was calculated using the Ct value of the *P.pastoris* genomic DNA and a standard curve. The *BspPel* gene copy number in the *P. pastoris* genome was determined with the following equation:

(1) $$\begin{array}{c} \mathrm{BspPel}\mathrm{gene}\mathrm{copy}\mathrm{number}=\\ \frac{\mathrm{BspPel}\mathrm{gene}\mathrm{copy}\mathrm{number}\mathrm{calculated}\mathrm{by}\mathrm{standard}\mathrm{curve}}{\mathrm{GAPDH}\mathrm{fragment}\mathrm{copy}\mathrm{number}\mathrm{calculated}\mathrm{by}\mathrm{standard}\mathrm{curve}} \end{array}$$

### Shaking Flask Culture and Fermentation in a 50-L Bioreactor

A single colony of each recombinant *P. pastoris* strain was transformed into 25 mL of BMGY and incubated for approximately 24 h at 30°C until the OD_600_ of the culture reached 2–6. The cells were then harvested by centrifugation (10,000 × *g* for 10 min at 4°C) and resuspended in 100 mL of BMMY in 250 mL baffled flasks to an OD_600_ = 1. Methanol was added into the medium every 24 h to a final concentration of 1.0% to maintain induction. Meanwhile, samples were taken every 24 h, and the pectate lyase activity was determined.

The fermentation inoculum of *P. pastoris* was prepared by cultivating the cells at 30°C with shaking at 250 rpm for 18–20 h in a 500 mL shaking flask containing 100 mL of YPD medium. Then, 10% (v/v) of the culture was inoculated into a 50 L fermenter (BIOTECH-3BG-7000A, Baoxing Co. Shanghai, China) with 30 L of fermentation basal salts medium (40 g L^–1^ glycerol, 22.7 g L^–1^ H_3_PO_4_, 0.93 g L^–1^ CaSO_4_, 18.2 g L^–1^ K_2_SO_4_, 14.9 g L^–1^ MgSO_4_⋅7H_2_O, 4.13 g L^–1^ KOH, and 7.0 g L^–1^ K_2_HPO_4_) and trace solution (12 mL). The trace solution consisted of 6 g L^–1^ CuSO_4_⋅5H_2_O, 0.08 g L^–1^ NaI, 3.0 g L^–1^ MnSO_4_⋅H_2_O, 0.2 g L^–1^ Na_2_MoO_4_⋅2H_2_O, 0.02 g L^–1^ H_3_BO_3_, 0.5 g L^–1^ CoCl_2_, 20 g L^–1^ ZnCl_2_, 65 g L^–1^ FeSO_4_⋅7H_2_O, 0.2 g L^–1^ biotin, and concentrated sulfuric acid (0.5% v/v). Samples were taken at regular intervals and analyzed for biomass, BspPel activity and total protein concentration. Each condition was measured in triplicate, and each experiment was investigated three times. The results are shown as the mean ± SD (standard deviation) from three independent experiments.

### Pectate Lyase Purification and Analysis

BspPel was obtained from the culture supernatant of *P. pastoris* cells harboring pA_m_NS-4BspPel with 6× HIS, The pectate lyase was purified on a Ni^2+^ nitriloacetic acid metal-affinity column according to the manufacturer’s instructions (Qiagen, Hilden, Germany) under naturing conditions. The recombinant pectin lyase in the supernatant was analyzed by SDS-PAGE, which was conducted using a 6% stacking gel and a 12% separating gel on a vertical mini gel apparatus (Bio-Rad, United States). The glycosylation sites were predicted by NetNGlyc 1.0 Server^2^. Protein marker was purchased from Fermentas (Burlington, Canada). Samples were mixed equally with 5× loading buffer and heated at 100°C for 5 min before electrophoresis. Proteins were stained with Coomassie Brilliant Blue R-250 (Amresco, Solon, OH, United States). The total protein concentration was determined by the Bradford method, using BSA (bovine serum albumin) as a standard.

### Measurement of Pectate Lyase Activity

Pectate lyase activity was routinely determined by measuring the absorbance change at 235 nm with 2 mg of PGA mL^–1^ as the substrate in 50 mM glycine-NaOH (pH 10.0) buffer containing 1 mM CaCl_2_ for 10 min. One unit (U) of pectin lyase activity was defined as the amount of enzyme that is required to produce unsaturated oligogalacturonide equivalent to 1 μmol of unsaturated digalacturonide min^–1^ using a molecular extinction coefficient of 4600 M^–1^ cm^–1^ at 235 nm.

### Biochemical Characterization of BspPel

The optimum pH of BspPel was determined at 80°C for 10 min, using different 0.05 M buffer systems: 50 mM NaH_2_PO_4_-Na_2_HPO_4_ (pH 6-8), 50 mM Gly-NaOH (pH 9-10), 50 mM Na_2_HPO_4_-NaOH (pH 11-12), with PGA as a substrate. Optimal temperature was determined by examing the activity of the enzyme in a 50 mM Gly-NaOH buffer (pH 10.0).

The effects of various metal ions and chemicals on activity were measured at final concentrations of 0.5 mM/1 mM or 1%/5% (v/v) into the reaction system. The degree of inhibition or activation of enzyme activity was measured as a percentage of enzyme activity of the control sample under standard reaction conditions.

The kinetic parameters of BspPel were determined for PGA, and the concentration varied from 0.1-8 mg/mL. The data were plotted according to the method of Lineweaver and Burk (Kluskens et al., 2003).

To investigate the thermal stability of the enzyme, purified BspPel was preincubated in the absence of substrates at 30–70°C, pH 10.0. Samples were taken at 30 min intervals over 4 h. Meanwhile, the enzyme was incubated in different buffers ranging from pH 3 to 11 for 16 h at 25°C to detect pH stability. The residual activities were measured at 80°C for 10 min using the standard pectate lyase assay method. These experiments were performed in triplicate. The statistical analyses of the experimental data were done with Microsoft Excel.

### Enzymatic Ramie Degumming by BspPel

Degumming was performed by a modified method described previously (Zhang et al., 2013; Zhou et al., 2017a). Dried and decorticated fibers were directly treated by enzyme or NaOH without boiling. A total of 1.5 g of ramie fibers was treated with 50 U mL^–1^ enzyme mixed with 30 mL of 50 mM glycine-NaOH buffer (pH 10.0) at 50°C for 4 h, which designated the enzyme method. When the enzyme-chemical method was used, enzyme-treated fibers were directly immersed in 30 mL of 0.5% (w/v) NaOH solution and subsequently treated at 120°C for 30 min. Beating and washing were performed to remove residual gum from the surface and wash with water. A drying process at 100°C was performed to obtain a constant weight to calculate the weight loss. Ramie fibers treated only by buffer at 50°C for 4 h and treated by 0.5% (w/v) NaOH solution at 120°C for 30 min were set as the negative control and positive control, respectively. All the measurements were performed three times.

Scanning electron microscope (SEM) was used to observe the surface morphology and microstructure of the treated ramie fibers.

## Conclusion

High-production of BspPel was achieved in *P. pastoris* with combined strategies, showing the yeast can be a desirable expression platform for pectate lyases. Due to good degumming efficiency and excellent biochemical characteristics, especially the remarkable stability under acid-to-alkaline pH and thermal conditions, BspPel was an efficient candidate for bio-degumming industry. Meanwhile, it provided a potential advantage in the kraft-pulp and detergent manufacturing industries, which require the use of high pH.

## Data Availability Statement

All datasets presented in this study are included in the article/Supplementary Material.

## Author Contributions

SL, YL, and XZ participated in the project design. XZ and YZ carried out the sequence analysis, promoter, gene cloning. XZ, XL, and CL carried out *P. pastoris* transformation, enzyme determination and the data analysis. XZ and SL coordinated the project and wrote the final manuscript. All authors contributed to the article and approved the submitted version.

## Conflict of Interest

The authors declare that the research was conducted in the absence of any commercial or financial relationships that could be construed as a potential conflict of interest.

## Footnotes

1 http://www.cbs.dtu.dk/services/SignalP/

2 http://www.cbs.dtu.dk/services/NetNGlyc
