# Supplementary material for: High-Level Expression and Biochemical Properties of A Thermo-Alkaline Pectate Lyase From Bacillus sp. RN1 in Pichia pastoris With Potential in Ramie Degumming
Source: Front Bioeng Biotechnol. 2020 Jul 24;8:850. doi: 10.3389/fbioe.2020.00850 (PMC7396651; doi:10.3389/fbioe.2020.00850)
Supplement: Supplementary file 1 [file Image_1.pdf]

Supplementary Figures

```
BspPel     1 MKKLISIIFIFVLGVVGSLTAAVSAEAASALNSGKVNPLADFSLKGFAAL
BliPelA    1 MKKLISIIFIFVLGVIGSLTAAVSAEAASSLSSGKVNPLADFSLKGFATL

                                                   N
BspPel    51 NGGTTGGEGGQTVTVTTGDQLIAALKNKNANTPLKIYVNGTITTSNTSAS
BliPelA   51 NGGTTGGEGGQTVTVTTGDQLIAALKNKNANTPLKIYVNGTITTSNTSAS

                     N
BspPel   101 KIDVKDVSNVSIVGSGTKGELKGIGIKIWRANNIIIRNLKIHEVASGDKD
BliPelA  101 KIDVKDVSNVSIVGSGTKGELKGIGIKIWRANNIIIRNLKIHEVASGDKD

BspPel   151 AIGIEGPSKNIWVDHNELYHSLNVDKDYYDGLFDVKRDAEYITFSWNYVH
BliPelA  151 AIGIEGPSKNIWVDHNELYHSLNVDKDYYDGLFDVKRDAEYITFSWNYVH

                             N
BspPel   201 DGWKSMLMGSSDSDNYNRTITFHHNWFENLNSRVPSFRFGEGHIYNNYFN
BliPelA  201 DGWKSMLMGSSDSDNYNRTITFHHNWFENLNSRVPSFRFGEGHIYNNYYN

BspPel   251 KIIDSGINSRMGARIRIENNLFENAKDPIVSWYSSSPGYWHVSNNKFVNS
BliPelA  251 KIIDSGINSRMGARIKIENNLFENAKDPIVSWYSSSPGYWHVSNNKFVNS

BspPel   301 RGSMPTTSTTTYNPPYSYSLDNVDNVKSIVKQNAGVGKINP
BliPelA  301 RGSMPTTSTTTYNPPYSYSLDNVDNVKSIVKQNAGVGKINP
```

Supplementary Figure 1. The sequence alignment of BspPel with BliPelA. Numbering begins at the N-terminus of the proteins. Identical amino acids are highlighted by black, while gaps are indicated by gray. Predicted N-glycosylation sites of BspPel are denoted by N symbol.

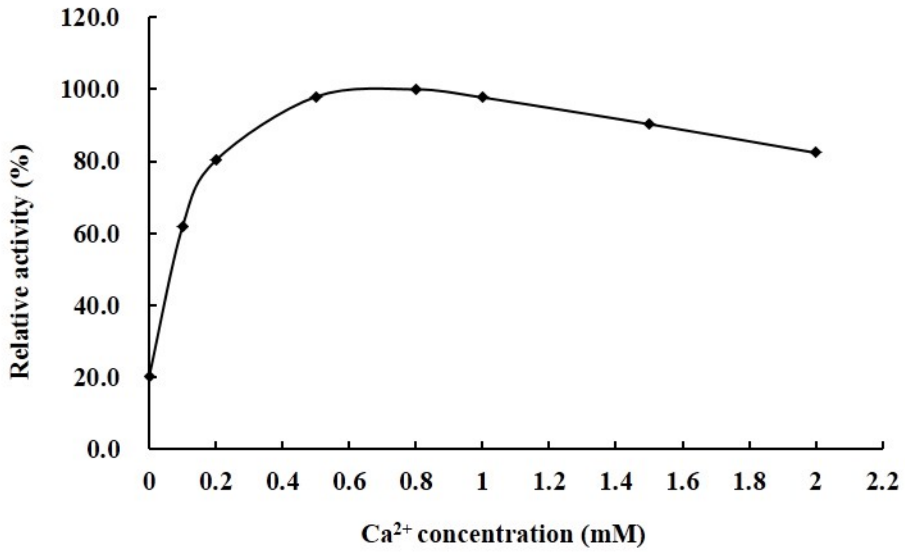

Supplementary Figure 2. Effect of Ca^{2+} on the activity of BspPel**.** Enzyme activity of 310 U/mL corresponds to 100% relative activity.
